# Modified Bain's Circuit as an Alternate to Non-invasive Ventilation in COVID 19

**DOI:** 10.7759/cureus.22772

**Published:** 2022-03-02

**Authors:** Rakesh B Singh, Prashant Mishra, Amit K Singh, Dhiraj K Srivastava, Varsha Gupta, Deepak Malviya, Shubham Rai

**Affiliations:** 1 Anesthesiology, Uttar Pradesh University of Medical Sciences, Etawah, IND; 2 Anesthesiology, Rama Medical College and Hospital, Ghaziabad, IND; 3 Preventive Medicine, Uttar Pradesh University of Medical Sciences, Etawah, IND; 4 Anesthesiology and Critical Care, Dr. Ram Manohar Lohia Institute of Medical Sciences, Lucknow, IND

**Keywords:** non-invasive ventilation, fresh gas flow, bipap, bain’s circuit, covid 19

## Abstract

Introduction

COVID-19 is a pandemic that severely affects the lungs. Symptomatically affected individuals often become severely hypoxic, requiring non-invasive ventilation. The scarcity of resources in resource-compromised countries like India led to the adoption of novel strategies like using Bain’s circuit for assisting spontaneous ventilation. This study compares the outcome when a standard circuit is replaced with a shortened Bain’s circuit.

Aims and objectives

To compare shortened Bain’s circuit and bilevel positive airway pressure (BiPAP) in spontaneously ventilated COVID 19 patients with regards to effects on hemodynamic stability and efficacy of ventilation using blood gas analysis.

Methodology

Twenty-four COVID patients aged between 35-70 years, requiring non-invasive ventilation but not tolerating BiPAP or not improving on BiPAP were enrolled in the study. Baseline heart rate and arterial blood gases (ABG) were recorded. Patients were then ventilated using shortened Bain’s circuit. Heart rate and ABG were then recorded two hours after ventilation.

Results

Hemodynamic and blood gas parameters were comparable between the two groups at baseline and on BiPAP. Group A showed better hemodynamic and blood gas profiles compared to group B, but the difference was not statistically significant because of small sample size.

Conclusion

Shortened Bain’s circuit may be a viable alternative to non-invasive ventilation in spontaneously breathing hypoxic patients with efficacy comparable to a standard Bain’s circuit and reduced chances of carbon dioxide retention. Studies with a larger sample size are needed to further validate the conclusion.

## Introduction

Since its outbreak in late 2019, COVID 19 has spread worldwide, leaving no country unscathed. The deadliest pandemic to hit the world in the last century has put the entire health care infrastructure on the verge of collapse in developed and developing countries alike. India reported its first case of COVID on 30 January 2020, and the case tally has been growing ever since [1,2]. A momentary relief in early 2021 was followed by a sudden surge of cases from March 2021, termed as ‘the second wave,’ which proved to be far more infectious and deadly than the previous year. This was attributed mostly to the emergence of various mutant strains. Unlike the first wave, which mostly affected the elderly and people with comorbidities, the second wave proved equally disastrous for young patients as well. The disease characterized by respiratory symptoms has ramped up its pace and is taking far less time to develop into full-blown acute respiratory distress syndrome (ARDS) and respiratory failure, requiring oxygen therapy and mechanical ventilation. At the time of inception, the consensus for the management of such cases was endotracheal intubation followed by the initiation of invasive ventilation. However, this modality was faced with challenges of its own. Trials in Italy [3,4,5] reviewed the use of non-invasive ventilation and were met with better outcomes when compared to their invasive counterparts when used early and for a short duration. Continuous positive airway pressure (CPAP), bilevel positive airway pressure (BiPAP), and high-flow nasal oxygen (HFNO) emerged as forbearers of non-invasive ventilation (NIV). Compared to invasive ventilation, they required less technical and logistical expertise and were able to prevent advancement to invasive modes in a considerable fraction of patients, decreasing the overall cost of treatment and mortality. BiPAP is being used extensively for the management of hypoxic patients. The use of different pressure gradients during inspiration and expiration has been a time-tested process in chronic obstructive pulmonary disease (COPD) patients and is proving its worth in the COVID era as well. Just like any other form of NIV, BiPAP requires a conscious, oriented, spontaneously breathing, and cooperative patient. To ensure the proper delivery of pressure to the patient, maintaining a proper seal with the patient’s airway is a must. This is achieved with specially designed masks, which form a seal around the nose and mouth or using a hood interface, with masks being more commonly used owing to affordability and availability. Achieving patient compliance with a mask in a COVID patient requiring prolonged therapy is a difficult task. Prolonged use may result in abrasions and necrosis on the bridge of the nose, chin, and cheek. Furthermore, highly pressurized gases escaping through leaks may escalate conjunctival dryness and discomfort. Another difficult task is encouraging the patient to synchronize their breaths with the pressure gradients generated by BiPAP. Often patients oppose the inspiratory pressures by closing their mouth and nostrils. The end result is ineffective ventilation and a high pressure generation, which is troublesome for the patient, often prompting them to refrain from the therapy. The overall result is failure to treat hypoxemia and having to move towards invasive ventilation, which may increase the cost and complexity of the treatment without guaranteeing a better outcome.

COVID 19 has baffled healthcare personnel worldwide and has led to the adoption of various novel medications and practices. One such practice that newly gained place in discussions was using Bain’s circuit attached to an NIV mask as an oxygen delivery device [6], wherein the fresh gas flow (FGF) is solely responsible for pressure gradient. The practice was met with considerable success with regard to improvement in oxygen saturation (SpO2). We conducted a pilot study on six COVID-19 patients who were ventilated using a standard full-length Bain’s circuit attached to a non-Invasive ventilation (NIV) mask. The pilot study showed promising results with regard to oxygenation and hemodynamic parameters. But, concerns regarding CO2 retention led to limited application. The present study aims to evaluate a modified Bains circuit, with length reduced to one-fourth of the original form.

## Materials and methods

The present study was conducted in COVID 19 hospital of Uttar Pradesh University of Medical Sciences after approval from the institutional ethical committee.

Inclusion and exclusion criteria

The study included patients who were confirmed to be COVID-19 positive by reverse transcription polymerase chain reaction (RT-PCR), were hypoxic with SpO2 <90% with a non-rebreathing mask and oxygen flow of 15 lit/min, and had intact neurological function with the ability to maintain airway. Patients who denied taking part in the study, who needed invasive ventilation, had a poor neurological status, had a history of respiratory disorders like emphysema or COPD, and had a history of cardiac or thyroid disorders were excluded from the study.

The study subjects included 24 COVID 19 patients who were hypoxic with SpO2 <90%, requiring non-invasive ventilation (NIV) using BiPAP. The patients fulfilled the basic criteria and had no contraindications for NIV, i.e., they were conscious, oriented to time/place/person and spontaneously breathing. Upon arrival in the hospital, baseline vital parameters including heart rate (HR), non invasive blood pressure (NIBP) and saturation of peripheral oxygen (SpO2) were recorded using a multi-channel monitor. Patients with oxygen saturation <90% on oximetry, not improving with non-rebreathing mask, necessitating an NIV were enrolled in the study after explaining the procedure and getting informed consent. A baseline arterial blood gas analysis was done. Patients were simultaneously put on pressure-controlled (PC)-BiPAP NIV mode using Drager Savina 300® (Draeger, Lübeck, Germany) ventilator, and patient-specific optimal settings were made. Patients were observed for 30 minutes and assessed thereafter. After 30 minutes, patients were randomly divided using sealed and numbered opaque envelop technique into two groups to be either spontaneously ventilated by a modified Bain’s circuit attached to an NIV mask or continued on BiPAP. The envelopes were opened by an anesthetist blind to the study.

Design

**Figure 1:**
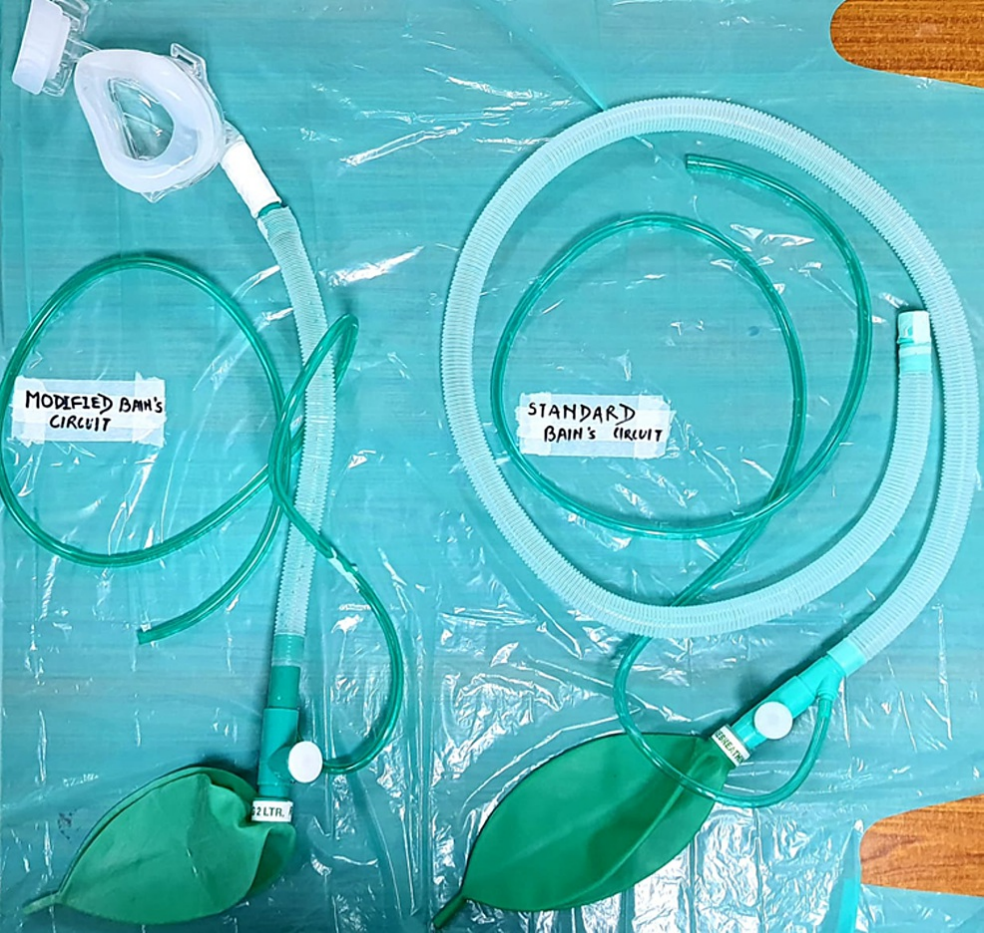
On the left is a modified Bain’s circuit with reduced length and attached NIV mask, on the right is a standard Bain’s circuit NIV - non-invasive ventilation

A standard factory-made Bain’s circuit was modified for the study. The standard Bain’s circuit which is 1.6 meters in length, was shortened. The outer corrugated plastic hose and inner polyvinyl chloride (PVC) tubing were cut at 40cm from an adjustable pressure limiting (APL) valve and reattached to the patient side connector. Special precautions were taken to prevent any leak in the tubing, and this was reaffirmed with standard tests before patient application. The position of the APL valve and two-liter reservoir bag remained unchanged. Figure 1 shows the circuit employed in the study compared to a standard full-length Bain’s circuit.

Group A patients were ventilated using the modified Bain’s circuit attached to an appropriately sized NIV mask. Group B patients were continued on ventilation using BiPAP.

In group A, fresh gas flow (FGF) in form of 100% oxygen was set at 10 liters/minute and the adjustable pressure limiting (APL) valve was completely opened. Hemodynamic variables were recorded after two hours. An arterial blood gases (ABG) test was done after two hours of ventilation, and partial pressure of CO2 (pCO2), partial pressure of O2 (pO2), and pH values were recorded. Patients not improving SpO2 or showing signs of deterioration in terms of the Glasgow Coma Scale (GCS) or hemodynamic variables were switched to invasive ventilation and excluded from the study.

Data collection and statistical analysis

All parameters, which included age, heart rate, SpO2, pO2, pCO2, pH, and SO2, were computed as means ± standard deviation and analyzed using the student’s unpaired T-test on open-source statistical software. A p-value of <0.05 was considered statistically significant.

## Results

Both groups contained six (50%) male and six (50%) female patients each.

**Figure 2:**
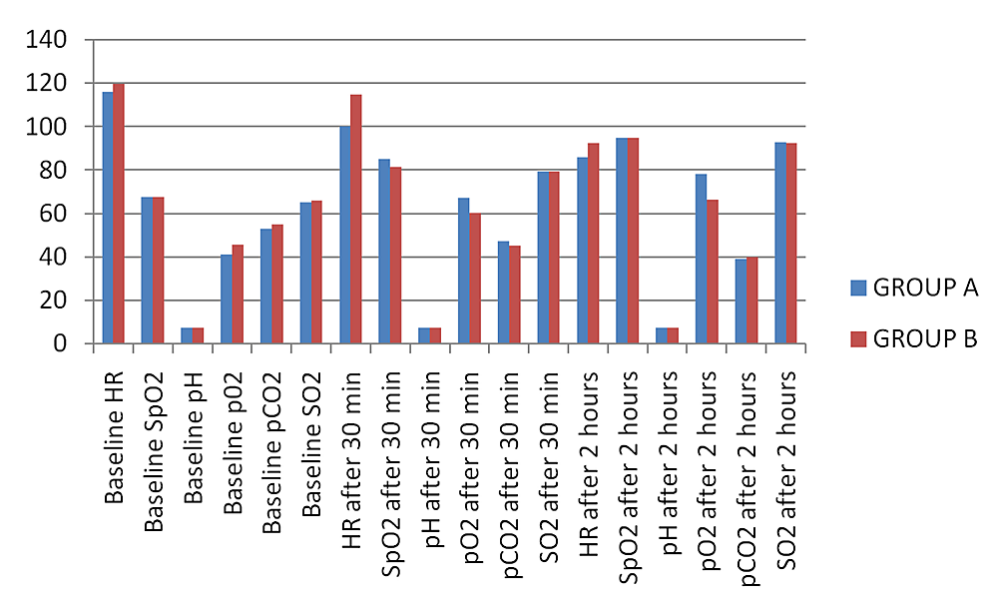
Comparison of study parameters among groups

Table 1 and Figure 2 show the intergroup comparison of study parameters including age, heart rate, SpO2, pO2, pCO2, pH, and SO2.

**Table 1:** Comparison of study parameters among groups BiPAP - bilevel positive airway pressure, df - degrees of freedom, HR - heart rate, Sp02 - oxygen saturation, pO2 - partial pressure of O2, pCO2 - partial pressure of CO2,

	Group A (modified Bain's circuit)		Ggroup B (BiPAP)		t-value	dF	p-value
	Mean	SD	Mean	SD			
Age (years)	52.58	9.41	51.83	12.60	0.17	22	0.87
Baseline HR (beats/minute)	116.08	33.27	119.83	30.16	0.29	22	0.78
Baseline SpO_2 _(%)	67.58	16.17	67.41	18.00	0.02	22	0.98
Baseline pH	7.33	0.08	7.30	0.07	0.98	22	0.34
Baseline pO_2 _(mmHg)	41.20	9.14	45.68	12.67	0.99	22	0.33
Baseline pCO_2 _(mmHg)	52.83	7.04	55.13	8.45	0.72	22	0.48
Baseline SO_2 _(%)	65.21	15.53	65.80	17.94	0.09	22	0.93
HR after 30 min (beats/minute)	100.25	24.87	114.83	28.71	1.33	22	0.20
SpO_2_ after 30 min (%)	85.00	5.70	81.33	10.71	1.05	22	0.31
pH after 30 min	7.37	0.08	7.32	0.06	1.73	22	0.10
pO_2_ after 30 min (mmHg)	67.05	12.14	60.30	17.30	1.15	22	0.26
pCO_2 _after 30 min (mmHg)	47.23	6.72	45.11	6.64	0.78	22	0.45
SO_2_ after 30 min (%)	79.26	8.03	79.33	9.81	0.02	22	0.98
HR after two hours (beats/minute)	85.92	8.03	92.50	8.40	1.96	22	0.06
SpO_2_ after two hours (%)	95.00	3.07	94.75	3.05	0.2	22	0.84
pH after two hours	7.42	0.06	7.38	0.07	1.5	22	0.15
pO_2_ after two hours (mmHg)	77.94	26.94	66.25	24.95	1.1	22	0.28
pCO_2_ after two hours (mmHg)	38.93	4.97	40.05	5.94	0.5	22	0.62
SO_2_ after two hours (mmHg)	92.66	25.95	92.52	4.08	0.02	22	0.98

Mean age in groups A and B were 52.58±9.41 years and 51.83±12.60 years, respectively.

The baseline heart rate (HR), pO2, SO2, SpO2, pCO2, and pH were comparable in both groups. Baseline HR was 116.08±33.27 per minute and 119.83±30.16 per minute in groups A and B, respectively. The baseline SpO2 was 67.58±16.17% and 67.41±18.00% in groups A and B, respectively. The baseline pH was 7.33±0.08 and 7.30V0.07 in groups A and B, respectively. The baseline pO2 was 41.2±9.14 mmHg and 45.68±12.67 mmHg in groups A and B, respectively. The baseline pCO2 was 52.83±7.04 mmHg and 55.13±8.45 mmHg in groups A and B respectively. The baseline SO2 was 65.21±15.53% and 65.80±17.94% in groups A and B, respectively.

The HR after 30 minutes was 100.25±24.87 per minute and 114.83±28.71 per minute in groups A and B, respectively. The SpO2 after 30 minutes was 85.00±5.70% and 81.33±10.71% in groups A and B, respectively. The pH after 30 minutes was 7.37±0.08 and 7.32±0.06 in groups A and B, respectively. The pO2 after 30 minutes was 67.05±12.14 mmHg and 60.30±17.30 mmHg in groups A and B, respectively. The pCO2 after 30 minutes was 47.23±6.72 mmHg and 45.11±6.64 mmHg in groups A and B, respectively. The SO2 after 30 minutes was 79.26±8.03% and 79.33±9.81% in groups A and B, respectively. The heart rate (HR), pO2, SO2, SpO2, pCO2and pH in both groups at 30 min were comparable.

The heart rate after two hours of ventilation was 85.92±8.03 per minute and 92.5±8.4 per minute in groups A and B, respectively. The SpO2 after two hours was 95±3.07% and 94.75±3.05% in groups A and B, respectively. The pH after two hours was 7.42±0.06 and 7.38±0.07 in groups A and B, respectively. The pO2 after two hours was 77.94±26.94 mmHg and 66.25±24.95 mmHg in groups A and B, respectively. The pCO2 after two hours was 38.93±4.97 mmHg and 40.05±5.94 mmHg in groups A and B, respectively. The SO2 after two hours was 92.66±25.95 mmHg and 92.52±4.08 mmHg in groups A and B, respectively.

After two hours of ventilation, group A shows numerically lower values of heart rate and pCO2 as compared to group B. Similarly, group B has numerically lower values of SpO2, pH, pO2, and SO2 compared to group A, after two hours of non-invasive ventilation

Although numerically different, statistical significance could not be found between the study groups in any parameter. This can be attributed to the considerably low sample size. However, patients ventilated using modified Bain's circuit showed stable hemodynamics and blood gas values.

## Discussion

Since its introduction in 1972 by Bain and Spoerel [7], Bain's modification of Mapleson's breathing circuit has been widely used and is considered the most efficient system during controlled ventilation [8]. It can be safely used for spontaneous ventilation as well, provided the fresh gas flow (FGF) is kept at 1.5 to 2 times per minute ventilation. Light-weight, low resistance, suitability for both spontaneous and controlled ventilation are among its advantages, placing it close to the criteria for an ideal breathing system. On looking at the functional analysis of Bain's circuit, many factors seem to affect the composition of inspired gas mixture. These include FGF, respiratory rate, expiratory pause, and tidal volume [8]. FGF greater than 1.5 times per minute ventilation is required to prevent rebreathing from the corrugated tubing. A standard Bain's circuit is 1.6 meters long, with the diameter of the outer corrugated tubing being 22 mm and the inner tubing being seven mm. Various studies [9,10] have been done to analyze its efficiency after increasing its length. However, no study has ever been conducted on shortening the circuit, to the best of our knowledge.

Sweeting et al. [9], in their study, compared circuits of length 1.6m and 9.6m and concluded that static compliance of the Bain system increased in proportion to its length. This translated into an extra gas requirement to achieve similar pressures. The 9.6m Bain system resulted in a statistically significant decrease in distal peak inspiratory pressures and tidal volumes in all cases when compared with the 1.6m Bain system with similar peak ventilator pressures. In our study, spontaneous ventilation was studied, making the patient responsible for the generation of inspiratory pressures. Hence, the smaller version of the Bain's circuit has an advantage over its longer counterpart in decreasing the work of breathing, resulting in better tolerance and decreased chances of fatigue in an already compromised respiratory system by decreasing the resistance. A shorter system also comes with the advantage of reduced FGF requirements. The reduced length of the breathing system reduces the amount of FGF to effectively flush the corrugated tubing of CO2 and decreases the chances of rebreathing. Even a modest reduction in oxygen requirements without compromising patient safety is crucial at this stage when the whole country is facing medical oxygen crisis that has resulted in a significant toll on human life.

Sellers et al. [10] studied the effect of using a two-meter and three-meter Bain system during spontaneous preoxygenation and concluded that the smaller system was easier to breathe through and needed less negative pressure distally on inspiration.

Kumari et al. [6] published a case study about using a full-length Bain's circuit as a continuous positive airway pressure device in a postoperative COVID-19 associated mucormycosis patient with type 1 respiratory failure. They used a fresh gas flow of eight liters/minute with the APL valve slightly closed. We have used a similar technique with a few alterations. The fresh gas flow was increased to 10 liters/minute, and the APL valve was left completely open to ease spontaneous ventilation without any increased risk of hypercarbia.

Our study demonstrates numerically superior values of HR, SPO2, PO2, PCO2, pH, and SO2 in patients ventilated using shortened Bain's circuit when compared to patients on BiPAP. Lower heart rate may be considered as an indicator of patient comfort. However, this could not be translated into statistical significance because of the small sample size.

In our study, the use of a further shortened system resulted in a reduced workload of COVID patients, whose work of breathing has already been exaggerated due to the disease. Another factor influencing the composition of inspired gas mixture is expiratory pause. In a patient with COVID-19, respiratory symptoms often lead to tachypnea, translating into diminished expiratory pause. This will contribute to a rise in the concentration of inspired CO2 unless considerably high FGF is used to flush out the expired gases. Reduction in circuit length positively impacts the economy of fresh gas flow required when the APL valve is fully open.

Rapid deterioration of patients from respiratory symptoms, warranting mechanical ventilation, had put significant pressure on developing and developed countries alike. BiPAP ventilation seems to have positively impacted patient prognosis by reducing the need for invasive ventilation where hypoxemia was not very severe. But the virtues of this modality are often outweighed by patient discomfort. Availability and technical expertise in low-cost settings are also a matter of concern. In both these situations, the shortened version of Bain's system may be used as a last resort to prevent mortality and morbidity. Better gas economics, lower infrastructure requirements, and better patient tolerance without compromising patient hemodynamics and blood gas variables make it a suitable alternative to NIV.

## Conclusions

The present study though devoid of statistical significance, demonstrates the effectiveness of a shortened Bain’s circuit as a viable alternate to BiPAP as an oxygen delivery device in hypoxic patients, which is well tolerated by patients. Patients ventilated using shortened Bain’s circuit showed numerically superior values of heart rate, pH, pO2, pCO2, and SO2. Improvement in the status of oxygenation and ventilation parameters translates into the viability of the design as an oxygen delivery device for patients requiring non-invasive ventilation. Studies with a larger sample size will be required to further validate the application of Bain’s circuit with reduced length in spontaneously breathing patients requiring non-invasive ventilation in COVID and non-COVID illness alike. If validated, this study might be a great boon for the health care infrastructure of developing and developed countries alike.
